# Disseminated Herpes Zoster in a Young Immunocompetent Adult

**DOI:** 10.7759/cureus.105467

**Published:** 2026-03-18

**Authors:** Sana Altaf, Oluwatito Roberts, Valerie Foy, Viktoriya Voytovych, Hisham Abbas, Mariam Dabaghyan

**Affiliations:** 1 Medicine, Touro College of Osteopathic Medicine, New York, USA; 2 Dermatology, Ross University School of Medicine, Saint Michael, BRB; 3 Dermatology, St. John’s Episcopal Hospital, New York, USA; 4 Internal Medicine, St. John's Episcopal Hospital, New York, USA

**Keywords:** acute kidney injury (aki), ‏acyclovir, disseminated cutaneous zoster, immunocompetent adult, varicella-zoster virus

## Abstract

Disseminated cutaneous herpes zoster (DCHZ), defined as lesions involving three or more dermatomes, is strongly associated with immunocompromised states and remains exceptionally rare in young immunocompetent adults. We present a case of DCHZ in a previously healthy 25-year-old male from Guatemala who presented with an eight-day history of a painful vesicular rash involving the right V2/V3, C3-C5, and T2-T3 dermatomes. Comprehensive evaluation for occult immunocompromise, including HIV testing, complete blood count, hemoglobin A1c, and urinalysis, was unrevealing. Treatment with high-dose intravenous acyclovir was complicated by acute kidney injury after four doses, with creatinine rising from 0.80 mg/dL to 2.60 mg/dL. Renal function improved following discontinuation of IV acyclovir and transition to oral valacyclovir. This case highlights the rarity of disseminated zoster in young immunocompetent adults and underscores the importance of targeted evaluation for underlying immunologic vulnerability and thorough investigation of acute kidney injury in the setting of DCHZ.

## Introduction

Varicella-zoster virus (VZV) reactivation typically presents as localized herpes zoster confined to a single dermatome and occurs most commonly in older adults or individuals with impaired cell-mediated immunity [1,2]. Disseminated cutaneous herpes zoster (DCHZ), defined as lesions involving three or more dermatomes, is strongly associated with immunocompromised states such as malignancy, HIV infection, or medication-induced immunocompromise [1,3,4]. Reports of DCHZ in immunocompetent hosts remain rare, particularly among younger adults [3-5]. High-dose intravenous acyclovir is the standard treatment for disseminated disease but carries a recognized risk of nephrotoxicity, which may occur early in the treatment course [2,3,6]. We present a case of DCHZ in a previously healthy 25-year-old man without identifiable immunocompromise, complicated by acute kidney injury (AKI) following brief acyclovir exposure.

## Case presentation

A 25-year-old male who migrated from Guatemala four years ago with no significant past medical history presented to the emergency department with an eight-day history of a painful, pruritic vesicular rash involving the right side of the face, neck, and chest. He reported fever and “flu-like” symptoms prior to rash onset. He denied similar prior rashes, recent travel, medications, or known immunocompromising conditions. He reported receiving all childhood vaccinations per the Guatemalan immunization schedule. Vital signs were normal on presentation, and he remained afebrile and hemodynamically stable throughout hospitalization.

**Figure 1:**
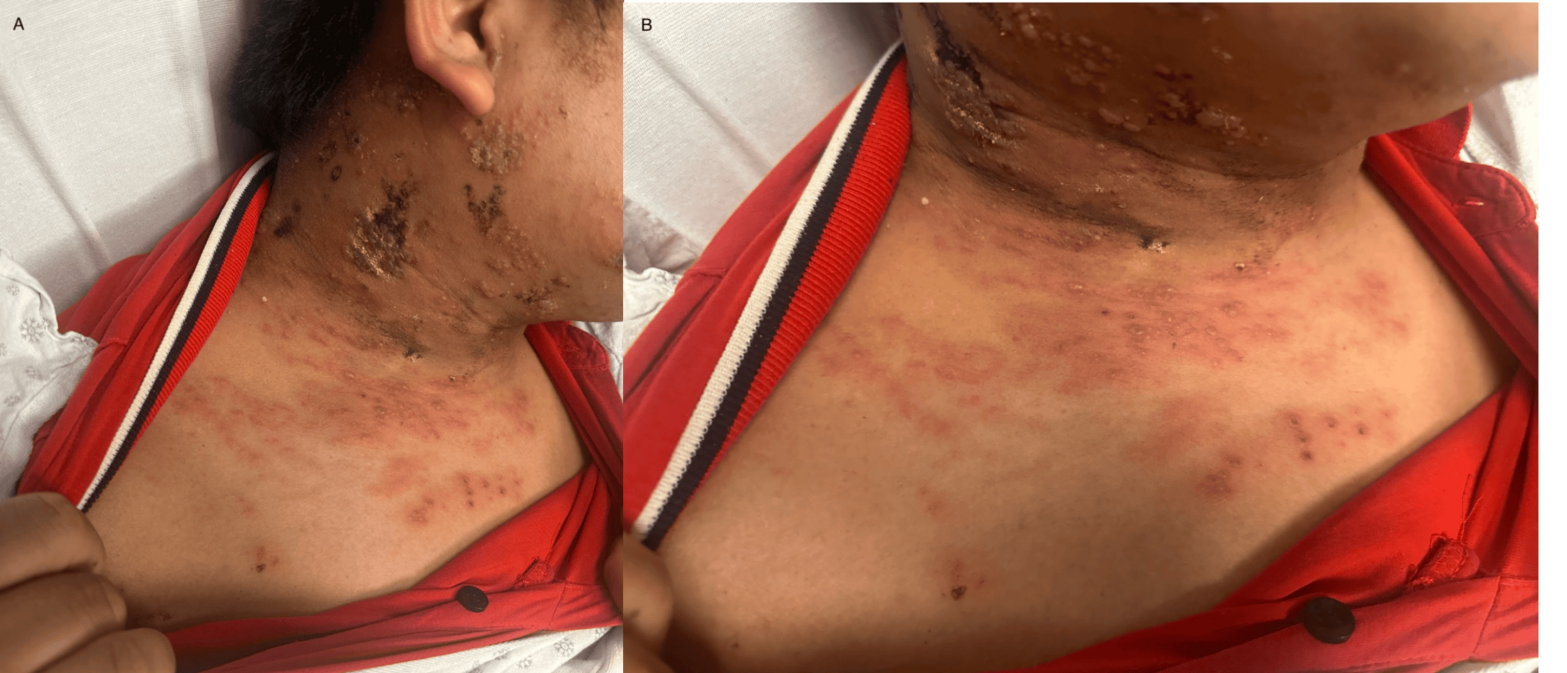
Disseminated cutaneous herpes zoster upon presentation to the hospital. (A, B) Multiple vesicles on an erythematous base with crusting in a unilateral dermatomal distribution involving the right V2/V3, C3-C5, and T2-T3 dermatomes.

**Figure 2:**
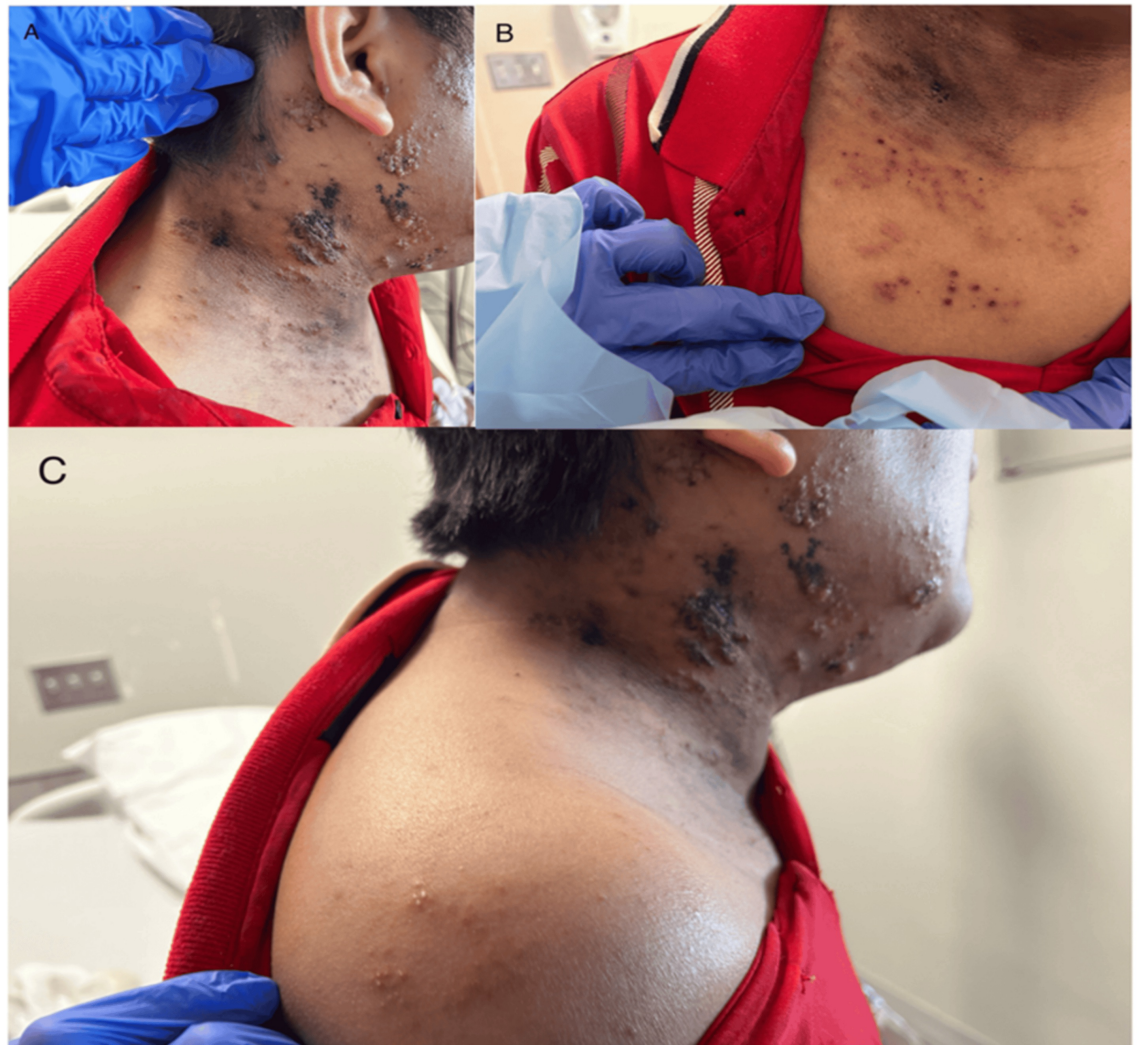
Clinical progression of disseminated cutaneous herpes zoster 24 hours after initial presentation with (A) right face and neck showing progression of grouped vesicles in V2/V3 and C3-C5 dermatomal distributions, (B) right anterior chest demonstrating vesicular lesions in the T2-T3 distribution, and (C) new vesicles forming on right shoulder.

On physical examination, grouped vesicles on an erythematous base with ulceration and necrosis were noted in a dermatomal distribution involving the right maxillary (V2) and mandibular (V3) divisions of the trigeminal nerve and the right C3-C5 dermatomes without crossing the midline. Numerous additional discrete vesicles were present on the anterior chest in the right T2-T3 distribution (Figures 1, 2). The patient denied any changes with hearing or ear pain. Given the presence of >20 vesicular lesions outside the primary and adjacent dermatomes, the findings were consistent with disseminated cutaneous herpes zoster. The patient was admitted for inpatient management, airborne and contact precautions were instituted, and dermatology was consulted.

Intravenous acyclovir was initiated on hospital day 0 at 10 mg/kg every 12 hours, with baseline creatinine 0.80 mg/dL and concurrent intravenous hydration. After four doses, creatinine increased to 1.40 mg/dL on day 1 and peaked at 2.60 mg/dL by day 2. IV acyclovir was discontinued and transitioned to oral valacyclovir 1,000 mg three times daily. Laboratory evaluation revealed normal complete blood count, liver function tests, hemoglobin A1c 5.5%, and urinalysis negative for protein, hematuria, or casts. Infectious evaluation included repeat HIV testing, which was negative by a fourth-generation antigen/antibody assay with HIV-1 RNA not detected. Serologic testing demonstrated herpes simplex virus (HSV)-1 IgG 44.5, HSV-2 IgG 0.7, VZV IgG 6.89, and VZV IgM 2.81, consistent with VZV. Creatinine improved by discharge on hospital day 7. The patient was instructed to continue valacyclovir 1,000 mg three times daily for four more days, totaling treatment for 10 days with gabapentin 600 mg once a day and topical lidocaine or capsaicin for post-herpetic neuralgia management.

## Discussion

Disseminated VZV is typically associated with impaired cell-mediated immunity, and reports in patients without recognized immunodeficiency remain uncommon, particularly among young adults [1]. Epidemiologic studies have identified age, immunosuppression, mechanical trauma, and psychological stress as risk factors for herpes zoster, although none were clearly identified in our patient [7]. A 2014 review identified only 28 cases of DCHZ in immunocompetent hosts at that time [3]. More recent publications continue to describe DCHZ in patients labeled “non-immunocompromised,” including occasional cases in younger patients [4]. The largest recent synthesis by Itagaki et al. combined 20 institutional cases with 42 previously published cases (n=62) with a median age of 71.5 years, highlighting that our 25-year-old patient represents a clear outlier [5]. In that cohort, in-hospital mortality was 0%, but postherpetic neuralgia occurred in ~40%, emphasizing that neurologic morbidity can be substantial despite low mortality [5]. Because DCHZ is defined as lesions involving multiple dermatomes outside the primary or adjacent ones, and our patient demonstrated involvement spanning at least five dermatomes, this supported disseminated disease rather than localized zoster [2,3]. This distinction triggers airborne and contact precautions until lesions crust and supports systemic antiviral therapy with closer monitoring [2].

Because DCHZ is unusual in a young adult, we pursued a focused evaluation for occult immunocompromise. Standard immunological evaluation includes HIV testing, complete blood count with differential, and serum immunoglobulin levels (IgG, IgA, IgM, IgE) [8]. The patient denied past history of taking systemic corticosteroids or biologics. HIV was excluded with repeat testing including HIV-1 RNA not detected. Additional screening supported immunocompetent status: no cytopenias, hemoglobin A1c 5.5%, liver-associated tests within normal limits, and urinalysis negative for protein, making nephrotic syndrome unlikely. The patient remained clinically stable without systemic symptoms, lymphadenopathy, or findings prompting malignancy workup.

A secondary issue was AKI following initiation of high-dose IV acyclovir. Drug-related renal injury occurs in 5%-21% of patients receiving IV acyclovir and typically manifests within the first few doses [9,10]. Acyclovir nephrotoxicity characteristically reverses with adequate hydration and dose reduction or discontinuation of the drug [11]. In our patient, the bland urinalysis without hematuria or proteinuria, normal liver tests, and improvement after discontinuing IV acyclovir and transitioning to oral valacyclovir supported a drug-associated process. However, causality cannot be proven definitively.

## Conclusions

Disseminated herpes zoster remains exceptionally rare in young immunocompetent adults. When identified, focused evaluation for occult immunocompromise is warranted, with malignancy screening reserved for patients with red-flag features. Our 25-year-old patient represents one of the youngest reported cases of disseminated cutaneous herpes zoster in an immunocompetent host, highlighting the need for further investigation into underlying genetic susceptibility factors.
